# Substernal goiter and laryngopharyngeal reflux

**DOI:** 10.1590/2359-3997000000266

**Published:** 2017-06-14

**Authors:** Mariana Gonçalves Rodrigues, Vergilius José Furtado de Araujo, Leandro Luongo de Matos, Flávio Carneiro Hojaij, Cesar Augusto Simões, Vergilius José Furtado de Araujo, Daniel Marin Ramos, Renata Lorencetti Mahmoud, Letícia de Moraes Mosca, Gustavo Borges Manta, Erivelto Martinho Volpi Lenine Garcia Brandão, Claudio Roberto Cernea

**Affiliations:** 1 Faculdade de Medicina Universidade de São Paulo São Paulo SP Brasil Faculdade de Medicina da Universidade de São Paulo (FMUSP), São Paulo, SP, Brasil; 2 Hospital das Clínicas FM USP São Paulo SP Brasil Disciplina de Cirurgia de Cabeça e Pescoço do Hospital das Clínicas da FMUSP, São Paulo, SP, Brasil; 3 Departamento de Anatomia FM USP São Paulo SP Brasil Departamento de Anatomia da FMUSP, São Paulo, SP, Brasil

**Keywords:** Substernal goiter, intrathoracic goiter, laryngopharyngeal reflux, gastroesophageal reflux, laryngitis

## Abstract

**Objective:**

This study aims to compare the prevalence of laryngopharyngeal reflux signs between two groups of patients undergoing thyroidectomy for voluminous goiter: substernal goiters and voluminous cervical goiter without thoracic extension.

**Subjects and methods:**

A retrospective case-control study was performed with data retrieved of the charts of the patients submitted to thyroidectomies occurred at a tertiary care center (Head and Neck Surgery Department, University of São Paulo Medical School) between 2010 and 2014. The selected thyroidectomies were allocated in two groups for study: patients with substernal goiters and patients with voluminous cervical goiter without thoracic extension. Cervical goiters were selected by ultrasonography mensuration. Clinical criterion was used to define substernal goiter.

**Results:**

The average thyroid volume in patients with substernal goiter was significantly greater than the average volume in patients with only cervical goiter (p < 0.001). The prevalence of signs of reflux laryngitis at laryngoscopy was significantly greater in substernal goiter patients (p = 0.036). Moreover, substernal goiter was considered as the unique independent variable for high reflux laryngitis signs at laryngoscopy (OR = 2.75; CI95%: 1.05-7.20; p = 0.039) when compared to only cervical goiter patients.

**Conclusion:**

This study shows a significant association between substernal goiters and signs of laryngopharyngeal reflux at preoperative laryngoscopy. Therefore, when compared with voluminous cervical goiters, the substernal goiters increase the chance of reflux laryngitis signs in patients.

## INTRODUCTION

Substernal goiter (SG) is the one whose extension exceeds the sternal notch towards the mediastinum. The diagnosis of substernal goiter depends on both clinical and radiological criteria (1,2). The published incidence of substernal goiter varies from 5% to 20%, probably due to a lack of a definitive definition (2). The symptoms and signs associated with a goiter depend on the size and location of the goiter (1), so direct compression of the goiter (mainly if substernal) against the trachea and the esophagus may be responsible for progressive dysphagia for solids, positional dyspnea and dysphonia. Surgery is the treatment of choice for SG (1-3) and requires proper planning. Complications related to surgery for substernal goiter are essentially similar to those of cervical goiter, such as bleeding, recurrent laryngeal nerve injury, hoarseness, and temporary or permanent hypoparathyroidism (1). Systematically preoperative vocal folds examination is recommended (1,2,4-6), because the quality and the functionality of the voice can suffer damage by both the compression of recurrent laryngeal nerve by the SG and the surgery itself (1). In additional, Holler and Anderson (7) recommend that all patients presenting with compressive thyroid symptoms should be evaluated for laryngopharyngeal reflux prior to performing a thyroidectomy, due to the association between these manifestations.

Laryngopharyngeal reflux (LPR) is an upper-esophageal manifestation of the gastroesophageal reflux disease (GERD), and is also a syndrome associated with symptoms including laryngitis, hoarse voice, chronic cough, and other complaints (8). LPR is believed to be caused by retrograde flow of gastric contents (particularly acid and pepsin) that affect pharyngeal and laryngeal mucosa by direct contact or as a physico-chemical stimulus for laryngeal spasm reflex mediated by the upper laryngeal, recurrent laryngeal and vagus nerves (9-12). The current gold-standard diagnostic test for LPR is dual-probe 24-hour pH monitoring or suggestive laryngoscopy associated with symptoms (9), whereas for GERD it is upper gastrointestinal endoscopy (UGIE) (8). Most patients with LPR do not exhibit GERD typical symptoms (regurgitation and heartburn), because laryngeal epithelium is more sensitive to gastric reflux than esophagus epithelium (13). As laryngeal mucosa is more sensitive, the frequency of reflux episodes and the reflowed volume of gastric content required to produce symptoms are much lower in the LPR when compared to GERD (9,13). Prevalence of LPR symptoms in patients with laryngeal diseases is considered as high as in general population (14,15).

Literature concerning goiter and laryngopharyngeal reflux is rare and presents poor or none substernal goiter series (7,16). Most of the published studies fails to achieve statistical significance to assert that the volume of thyroid interferes with the incidence of LPR.

This study aims to compare the prevalence of laryngopharyngeal reflux signs between two groups of patients undergoing thyroidectomy for voluminous goiter: substernal goiters (SG) and voluminous cervical goiter (CG) without thoracic extension.

## SUBJECTS AND METHODS

A retrospective case-control study approved by the institutional IRB (number 712/2011) was performed with data retrieved of the charts of the patients submitted to consecutive thyroidectomies occurred at a tertiary care center (Head and Neck Surgery Department, University of São Paulo Medical School) between 2010 and 2014. The selected patients were allocated in two groups for study: GROUP 1 consists of patients with SG, and GROUP 2 consists of patients with CG. Cervical goiters were selected by ultrasonography mensuration. At our Institution, clinical criterion is resorted for substernal goiter diagnosis (caudal extension of thyroid gland below the clavicles or sternal notch on physical examination), while radiological imaging tests determine mediastinal extension of substernal goiter. Goiters with volume at least 40 cc (around 2.5-fold the normal volume of the thyroid gland) were considered voluminous by our service. Patients in antireflux therapy were not selected for this study.

Fifty-two patients were selected for Group 1 (SG) and 61 for Group 2 (CG). The following parameters were studied from collected data: demographic data, thyroid function, preoperative compressive symptoms, thyroid gland volume measured by preoperative ultrasonography, vocal fold mobility and suggestive signs of reflux laryngitis in preoperative laryngoscopy (arytenoid hyperemia, laryngeal mucosa edema and vocal folds’ edema).

All laryngoscopies were performed by the same medical staff, using flexible laryngoscope Welch Allyn^®^ 3.4 mm with white light. The focus of vocal folds examination was to evaluate their functionality and identify possible injuries. Suggestive signs of reflux laryngitis were considered positive when were identified arytenoid hyperemia, laryngeal mucosa edema and vocal folds edema (posterior laryngitis).

### Statistical analysis

Distributions were defined as parametric by Kolmogorov-Smirnov test. The values obtained from the study of each parametric quantitative variable was organized and expressed as mean and standard deviation. Absolute and relative frequencies were used for qualitative variables. Frequency comparisons of a phenomenon between groups of qualitative variables were performed with application of chi-square test or Fisher’s exact test. Student’s t test was used for comparisons between the averages of two qualitative variables. The variables that present p < 0.2 in the univariate analyses were eligible for the multivariate analysis, in which the odds ratio (OR) and confidence interval 95% (CI95%) were calculated using logistic regression. In all analyzes the statistical program SPSS^®^ version 17.0 (SPSS Inc.^®^, Illinois, USA) was used and in all comparisons was adopted statistical significance level of less than 5% (p ≤ 0.05).

## RESULTS

Female patients predominated in both groups. There were 45 women in Group 1 and 55 in Group 2. Mean age was also similar between groups, and the sixth decade predominated in both of them. The groups were considered homogeneous and suitable to comparison (Table 1).

**Table 1:** Descriptive data of preoperative thyroidectomy patients with substernal or cervical goiter

Variable	Substernal goiter	Cervical goiter	P value
Age**	57.7 ± 11.2 years	51.6 ± 12.6 years	0.100*
Female	87.8%	90.2%	0.687*
Positive anti-thyroid peroxidase	6.1%	17.0%	0.117^†^
Positive anti-thyroglobulin	12.2%	18.8%	0.376*
Positive TRAB	4.1%	12.2%	0.239^†^
Preoperative cytology Bethesda I Bethesda II Bethesda III Bethesda IV Bethesda V	2.6% 81.6% 10.6% 5.2% -	1.8% 87.4% 7.2% 1.8% 1.8%	0.853*
Medication Levothyroxine Antithyroid drugs	5.9% 15.4%	15.4% 15.4%	0.403*
Nodules (ultrasonography) Unilateral Bilateral	22.9% 77.1%	14.6% 85.4%	0.296*
Goiter volume, cc (ultrasonography)**	163.0 ± 103.6	93,4 ± 61.1	**< 0.001^#^**
Compressive symptoms	85.7%	78.3%	0.322*
Laryngopharyngeal reflux signs in pre-operative laryngoscopy	41.5%	20.5%	0.036*
Abnormal vocal fold mobility in pre-operative laryngoscopy	5.0%	11.4%	0.437^†^
Pre-operative TSH (μU/mL)**	1.31 ± 1.20	114 ± 1.08	0.444^#^

* Chi-square test; ** Mean ± standard deviation; ^†^ Fisher’s exact test; ^#^ Student’s t test.

On preoperative period, 44 patients in Group 1 reported respiratory and/or digestive compressive symptoms, while 47 patients in Group 2 complained of these symptoms on the same period. The average thyroid volume of Group 1 was significantly higher than the other group’s average (respectively, 163.0 vs*.* 93.4 cc; p < 0.001 – Student’s *t* test). Suggestive reflux laryngitis signs diagnosed by preoperative laryngoscopy were also significantly higher in Group 1 (41.5% vs*.* 20.5%, respectively; p = 0.036). Thyroid function, measured by preoperative TSH value, was similar between the two groups. The results concerning autoantibody research, preoperative cytology, use of medication, as well as details about ultrasonography and laryngoscopy could also be found in Table 1.

In order to evaluate a possible confounding bias (the group with substernal goiter has higher reflux laryngitis signs but patients also had greater volume goiter), the goiter’s volume was stratified by the mean of the totality of patients (124.0 cc) and a multivariate analysis was performed (Table 2). Substernal goiter was considered as the unique independent variable for high reflux laryngitis signs at laryngoscopy (OR = 2.75; CI95%: 1.05-7.20; p = 0.039) when compared to only cervical goiter patients.

**Table 2:** Multivariate analysis for predictive factors for reflux laryngitis signs at laryngoscopy in patients with voluminous goiter

Variable	Odds ratio	Confidence interval 95%	P value*
Goiter volume ≤ 124.0 cc > 124.0 cc	1.00 (reference) 2.17	- 0.81 – 5.84	0.123
Patient’s groups Cervical goiter Substernal goiter	1.00 (reference) **2.75**	- **1.05 – 7.20**	**0.039**

* Logistic regression.

## DISCUSSION

The relationship between thyroid gland diseases and LPR is a recent subject in Head and Neck Surgery literature. This study shows a significant association between substernal goiters and LPR signs in preoperative laryngoscopy. Accordingly, when compared with large cervical goiters, substernal goiters provide a greater chance of signs of reflux laryngitis. To the best of our knowledge, this is the first study to present this relationship with a statistical significance level.

Although some authors (7,17) do report reflux laryngitis or LPR series when studying thyroid goiter, they are considered as concomitant diseases, in disagreement with the present findings. For instance, Fiorentino and cols*.* (17) attribute persistence of dysphagia and dysphonia after thyroidectomy to reflux disease, and concluded that both LPR and goiter compression to anatomical structures could cause such symptoms.

Nam and cols*.* (6) asserted that 35.8% of their preoperative thyroidectomy patients had some laryngeal disease diagnosed by laryngoscopy before surgery, of which 76% had LPR. Such results contribute to LPR relevance in patients undergoing thyroidectomy. In agreement with Nam and cols*.,* LPR occurrence is relevant in both Groups studied; however patients with substernal goiter presented significantly more to have signs of that condition.

Hamdan and cols. (16) found a positive relationship between thyroid goiter and LPR, and also that patients with nodular goiters were more likely to present LPR than patients with diffuse goiter, corroborating the findings of our study.

LPR symptoms may be present in 33.9% of patients undergoing thyroidectomy (7), consistent with general population prevalence (8). Nevertheless, among more symptomatic patients undergoing thyroidectomy, 68.4% of those who complain of dysphonia and 56% of those who complain with dysphagia have LPR signs and symptoms (7). This increase in percentage of symptomatic individuals was attributed to reflux by Holler and Anderson, which is an acceptable statement. Whereas LPR diagnosis may be based on suggestive laryngoscopy associated with symptoms (9), Holler and Anderson paper and the present one reinforce the association between LPR and thyroid disease, since the first paper studied symptomatic patients, while the present one valued laryngoscopic signs of LPR. Given the fact that the aim of Holler and Anderson’s study, obviously, was not the same as ours, it is possible to point out some differences between them. The first study has no SG sample and has lower thyroid mean volume (38.7 cc vs 163.0 cc for Group 1 and 93.4 cc for Group 2). This series thereby refutes the assumption that goiter and LPR are always concomitants by co-incidence, owing to the fact that intrathoracic extension of thyroid gland or its higher volume are significantly more associated with LPR than voluminous goiters restrict to the neck.

Usually the LPR is a chronic condition resulting from extra-esophageal repeated exposure to gastric reflux (10). Since the key symptoms of LPR are nonspecific, many patients undergo laryngoscopy (18). Once cancer is ruled out, diagnosis is given when there are erythema, edema, ventricular obliteration, postcricoid hyperplasia and pseudosulcus (18). Laryngoscopy and monitoring pH are the methods used for the diagnosis of LPR (13), though their real specificity and sensitivity values have been interrogated (18). Traditionally, successful empirical treatment with proton pump inhibitors is regarded as a diagnostic method (10); however this method has its limitations and is also questioned by the literature (18,19). In this study, the criteria used to diagnose “reflux laryngitis disease” were the following laryngoscopic findings: arytenoid hyperemia and laryngeal mucosa and the vocal folds edema (posterior laryngitis). Although such signs are very suggestive of reflux laryngitis, other hypotheses must be considered once laryngitis is identified, such as: post-nasal drip, sinusitis, allergies, pulmonary diseases, autoimmune diseases, granulomatous diseases and Reinke’s edema (10,11,18). Once the differential diagnostics have been mentioned, it is important to reinforce that both samples of patients were homogeneous in the present study, so even if some cases assumed as “reflux laryngitis” by examiner belong to other etiologies, their distribution among groups would be hypothetically homogeneous, therefore the findings statistical validity is preserved.

Taking into account that laryngopharyngeal mucosa is more sensitive than the esophageal; fewer exposures to contents of gastric reflux would be enough to cause upper-esophageal symptoms of reflux (9,13). Erickson and Sivasankar (20) stated that less than three reflux episodes per week could damage vocal folds mucosa, while zero to 50 reflux episodes per day are considered normal for distal esophagus. For that reason, it is reasonable to assume that whether esophagus peristalsis is impaired, an early manifestation of reflux could occur in upper-esophageal region, such as LPR.

**Figure 1:**
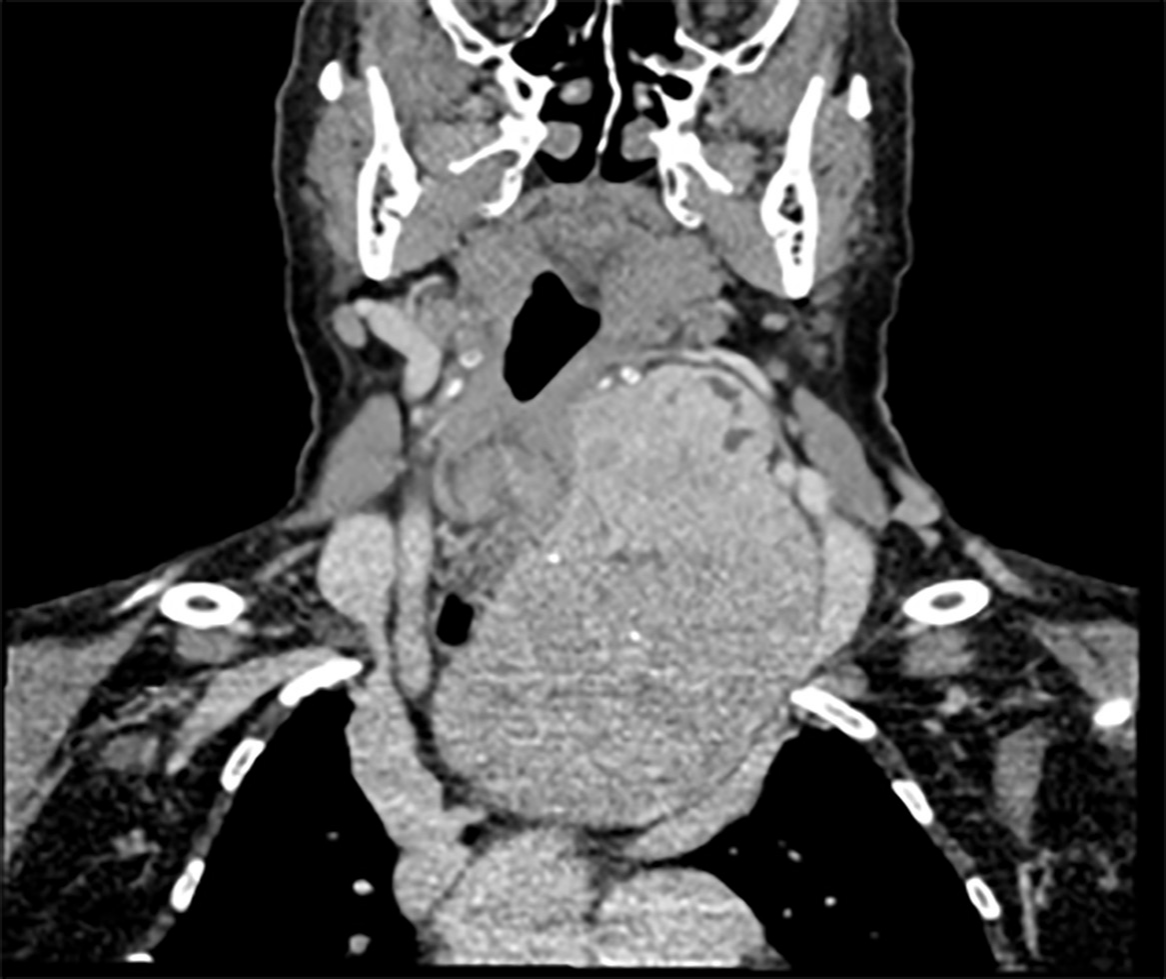
Preoperative CT of a patient with SG, showing a possible compression of the vagus nerve against the first rib.

The authors of this study believe that higher incidence of signs of reflux laryngitis in substernal goiters group may have an anatomical explanation. It is known that esophagus is innervated by both sympathetic and parasympathetic nerves. The parasympathetics control the peristalsis through the vagus nerve. The nerve fibers to striated muscles depart from the vagus in the upper part of the neck as the recurrent laryngeal nerve. In an extreme situation, a bilateral vagotomy above the origin of the pharyngoesophageal branches could cause abolishment of cervical esophagus peristalsis (21). The vagus nerve also produces excitatory and inhibitory stimuli in the lower esophageal sphincter (21). Therefore, esophageal peristalsis could be impaired if, somehow, the vagus nerve function was compromised. Theoretically, the vagus function could be altered if thyroid gland compresses the nerve against an anatomical structure (the first rib for example), while the inferior pole of the goiter expands and migrates toward thorax (Figure 1).

Another plausible hypothesis is that the direct compression of a voluminous goiter against digestive tract would cause an esophageal narrowing and so more food residue would accumulate in the upper part of the esophagus. When esophageal wall compliance is decreased, resistance to the movement of the bolus is increased, and a liquid bolus may flow back up through the ineffective contraction wave (21), accordingly a substernal goiter could alter esophagus function. Literature (22) also discloses that small physical stimuli in the hypopharynx can cause transient relaxation of the lower esophageal sphincter and thus enable the occurrence of reflux. Hypothetically, the largest food residue in upper-esophagus, secondary to goiter’s compression, could stimulate the hypopharynx and cause episodes of gastroesophageal reflux. As the average SG volume is higher than voluminous CG, it is expected that SG is more likely to cause esophageal stricture.

Evidently, these hypotheses have not a practical experimental support yet. The authors report those inferences from their surgical experience and theoretical study of cervical and thoracic anatomy. Moreover, the present series has substernal goiters up to 266.6 cc, which can cause direct compressive symptoms in different cervical structures such as the trachea (23,24), and the superior vena cava (25).

Several studies (7,17) recommend treatment of reflux before thyroid surgical approach, because whether the symptoms of cough, hoarseness and dysphagia were due to GERD, an indication of thyroidectomy can be reviewed depending on the original disease of the patient. As far as we know, this study is the first in the literature to show a direct relationship between the extent of intrathoracic goiter and higher incidence of LPR.

It is important to state some limitations regarding the present study. For instance, ultrasonography was used to evaluate goiter volume in both groups, but it is known that the intrathoracic portion could be underestimated by this method. Although laryngoscopies were not performed by the same person, they were performed by the same medical staff, whose professionals have equal training and the same diagnosis criteria for standardized possible findings, which include LPR signs. Also, in the present study, postoperative laryngoscopies data was not collected, therefore the authors could not analyze a possible change (or even an improvement) of LPR signs after thyroidectomy in patients who had substernal goiter. Neither dual-probe 24-hour pH monitoring nor patients’ symptoms were used for gastroesophageal reflux diagnosis, however, the present study found out indirect signs of LPR, and the occurrence of those signs was compared between the two groups. Also, the authors propose physiopathological explanations for greater occurrence of LPR signs in substernal goiter group. As well as dual-probe 24-hour pH monitoring, suggestive laryngoscopy associated with symptoms is known as another way to diagnose LPR, but the patients studied were not asked about suggestive symptoms. Nevertheless, the authors believe that despite those limitations, their anatomical hypothesis for greater incidence of LPR signs in people with substernal goiter may show a clinical relevance for future studies. Finally, there is also the possibility of LPR may be associated not only to intrathoracic extent of goiter, but also with the thyroid volume. However, to test this hypothesis, it would be required a study design that includes different thyroids sizes, and not only cases of voluminous goiters as analyzed in this study.

In conclusion, this study demonstrated that patients with substernal goiters have significantly higher incidence of laryngoscopic signs of laryngopharyngeal reflux compared with voluminous cervical goiter without thoracic extension and these patients must be investigated for this disease and specific treatment should be considered. This fact also creates opportunities for development of studies on the pathophysiology of this condition in patients with voluminous goiters with intrathoracic extension.
